# A geometric approach to characterize the functional identity of single cells

**DOI:** 10.1038/s41467-018-03933-2

**Published:** 2018-04-17

**Authors:** Shahin Mohammadi, Vikram Ravindra, David F. Gleich, Ananth Grama

**Affiliations:** 1 Computer Science and Artificial Intelligence Laboratory, MIT, Cambridge, MA 02139 USA; 2 Broad Institute of MIT and Harvard, Cambridge, MA 02142 USA; 3 Department of Computer Science, Purdue University, West Lafayette, IN 47907 USA

## Abstract

Single-cell transcriptomic data has the potential to radically redefine our view of cell-type identity. Cells that were previously believed to be homogeneous are now clearly distinguishable in terms of their expression phenotype. Methods for automatically characterizing the functional identity of cells, and their associated properties, can be used to uncover processes involved in lineage differentiation as well as sub-typing cancer cells. They can also be used to suggest personalized therapies based on molecular signatures associated with pathology. We develop a new method, called ACTION, to infer the functional identity of cells from their transcriptional profile, classify them based on their dominant function, and reconstruct regulatory networks that are responsible for mediating their identity. Using ACTION, we identify novel Melanoma subtypes with differential survival rates and therapeutic responses, for which we provide biomarkers along with their underlying regulatory networks.

## Abstract (web summary)

Functional characterisation of single cells is crucial for uncovering the true extent of cellular heterogeneity. Here the authors offer an approach to infer functional identities of cells from their transcriptomes, identify their dominant function, and reconstruct the underlying regulatory networks.

## Introduction

Complex tissues typically consist of heterogeneous populations of interacting cells that are specialized to perform different functions. A cell’s functional identity is a quantitative measure of its specialization in performing a set of primary functions. The functional space of cells is then defined as space spanned by these primary functions, and equivalently, the functional identity is a coordinate in this space. Recent advances in single-cell technologies have greatly expanded our view of the functional identity of cells. Cells that were previously believed to constitute a homogeneous group are now recognized as an ecosystem of cell types^1^. Within the tumor microenvironment, for example, the exact composition of these cells, as well as their molecular makeup, have a significant impact on diagnosis, prognosis, and treatment of cancer patients^2^.

The functional identity of each cell is closely associated with its underlying type^3^. A number of methods have been proposed to directly identify cell types from the transcriptional profiles of single cells^4–9^. The majority of these methods rely on classical measures of distance between transcriptional profiles to establish cell types and their relationships. However, these measures fail to capture weakly expressed, but highly cell-type-specific genes^10^. They often require user-specified parameters, such as the underlying number of cell types, which critically determine their performance. Finally, once the identity of a cell has been established using these methods, it is often unclear what distinguishes one cell type from others in terms of the associated functions.

To address these issues, we propose a new method, called archetypal-analysis for cell-type identification (ACTION), for identifying cell types, establishing their functional identity, and uncovering underlying regulatory factors from single-cell expression datasets. A key element of ACTION is a biologically inspired metric for capturing cell similarities. The idea behind our approach is that the transcriptional profile of a cell is dominated by universally expressed genes, whereas its functional identity is determined by a set of weak, but preferentially expressed genes. We use this metric to find a set of candidate cells to represent characteristic sets of primary functions, which are associated with specialized cells. For the rest of the cells, that perform multiple tasks, they face an evolutionary trade-off—they cannot be optimal in all those tasks, but they attain varying degrees of efficiency^11^. We implement this concept by representing the functional identity of cells as a convex combination of the primary functions. Finally, we develop a statistical framework for identifying key marker genes for each cell type, as well as transcription factors that are responsible for mediating the observed expression of these markers. We use these regulatory elements to construct cell-type-specific transcriptional regulatory networks (TRN).

We show that the ACTION metric effectively represents known functional relationships between cells. Using the dominant primary function of each cell to estimate its putative cell type, ACTION outperforms state-of-the-art methods for identifying cell types. Furthermore, we report on a case study of cells collected from the tumor microenvironment of 19 melanoma patients^12^. We identify two novel, phenotypically distinct subclasses of *MITF*-high patients, for which we construct the TRN and identify regulatory factors that mediate their function. These factors provide novel biomarkers, as well as potential therapeutic targets for future development.

## Results

**Fig. 1:**
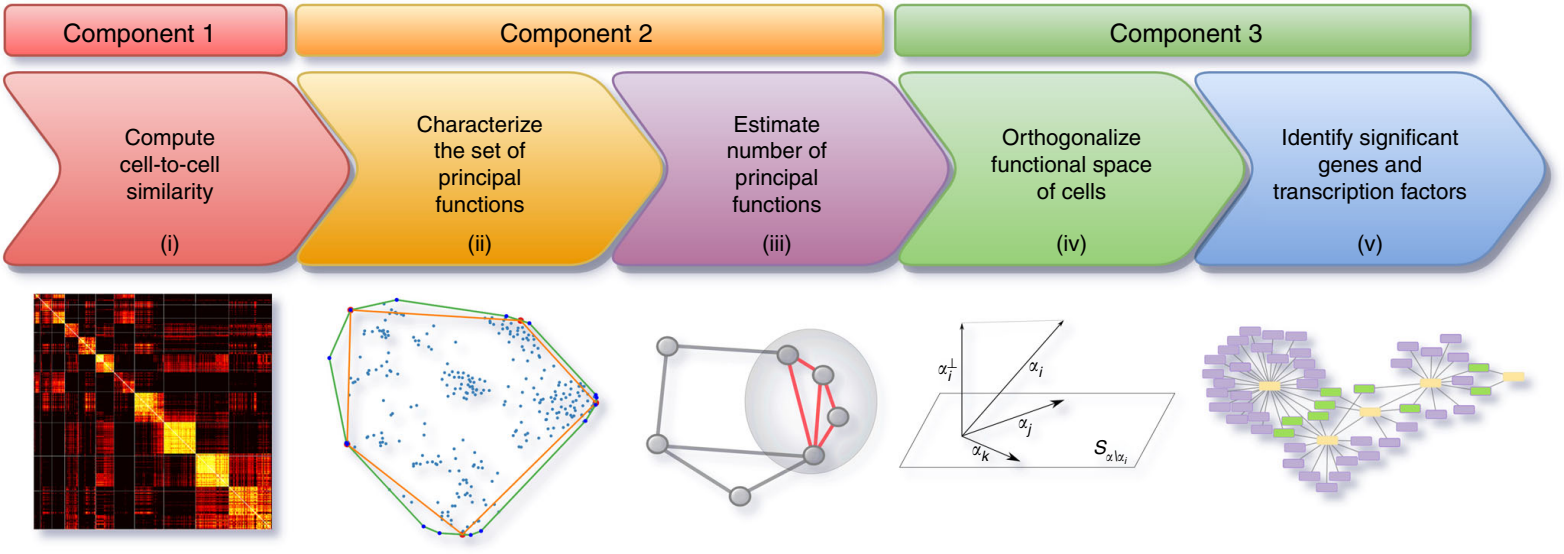
Overview of ACTION. ACTION consists of five main steps: **i** A biologically inspired metric to capture similarity among cells. **ii** A geometric approach for identifying the set of primary functions. **iii** An automated mechanism for identifying the number of primary functions needed to represent all cells. **iv** An orthogonalization procedure for identifying key markers for each primary function. **v** A statistical approach for identifying key regulatory elements in the transcriptional regulatory network. These steps are grouped into three main components in the ACTION method that are each discussed in the methods section

The ACTION framework consists of three major components, shown in Fig. 1: (i) a robust, yet sensitive measure of cell-to-cell similarity, (ii) a geometric approach for identification of primary functions, and (iii) a statistical framework for constructing cell-type-specific TRN. Our framework starts by defining a cell similarity metric that simultaneously suppresses the shared, but highly expressed genes and enhances the signal contributed by preferentially expressed markers. The next component of our method is a geometric approach for identifying primary functions of cells. Each of these primary functions is represented by a corner of the convex hull of points defined within the functional space of cells. We refer to these corners as archetypes and the functional identity of each cell is represented by a convex combination of these archetypes. Finally, ACTION uses a novel method to orthogonalize archetypes, find key marker genes, and assess the significance of each transcriptional factor in mediating the transcriptional phenotype associated with each archetype. Finally, we use this method to construct the characteristic TRN of each cell type. In what follows, we describe, validate, and discuss each component in detail.

### Representing functional relationships between single cells

**Fig. 2:**
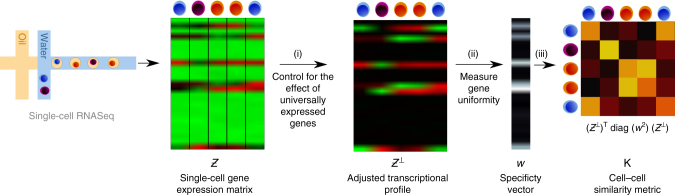
Workflow of ACTION cell-to-cell similarity metric. Computation of the ACTION metric consists of three main steps. i The effect of universally expressed genes is masked out to construct an adjusted transcriptional profile. ii A gene expression specificity vector is computed that assigns weights to each gene based on its informativeness. iii The ACTION kernel is computed as the weighted dot product of adjusted transcriptional vectors

A fundamental component of many methods for identifying cell types is a measure for quantifying the similarity between individual cells. Most prior methods rely on traditional measures, such as linear correlation, which are not specifically targeted towards transcriptomic profiles. In contrast, we define a similarity metric, or formally a kernel, specifically designed for measuring the similarity between single-cell transcriptomes^10^. Our approach is illustrated in Fig. 2 and the mathematical models underlying the metric are described in the Methods section, Computing ACTION metric. In summary, we first adjust the raw transcriptional profiles of cells to remove the effect of universally expressed genes by projecting them onto the orthogonal space relative to the universally expressed profile. We then boost the contribution of cell-type-specific genes using an information theoretic approach. The final similarity is then a weighted inner-product kernel between these adjusted profiles.

To establish the superiority of our metric, we compare it against an alternate measure specifically designed for single-cell analysis, SIMLR^13^. SIMLR combines a number of distance metrics to learn a joint similarity score that maximizes the block diagonal structure of the resulting matrix. We also compare ACTION with the normalized dot product resulting from two nonlinear dimension-reduction techniques: multidimensional scaling (MDS) and Isomap. While ACTION is a non-parametric method, the other methods have one or more parameters that need to be specified by the user. For SIMLR, we need to specify the true number of cell types. For all methods other than ACTION, we must specify the dimension of the low-dimensional subspace. To give them the best chance at competing with ACTION, we evaluate ten different values for the dimension of projected subspace (from 5 to 50 with increments of 5) and report the best results obtained over all configurations.

To assess the quality of computed similarities between cells, we used each metric with kernel *k*-means, starting from 100 different initializations, in order to comprehensively assess their ability to identify discrete cell types. We apply this technique to four different datasets (see Methods, Datasets). These datasets are derived from different single-cell technologies, have hundreds to thousands of cells, and span a wide range of normal and cancerous cells. We compare the predicted cell types against the annotated cell types in the original dataset using three different measures, namely Adjusted Rand Index (ARI), F-score, and Normalized Mutual Information (NMI).

**Fig. 3:**
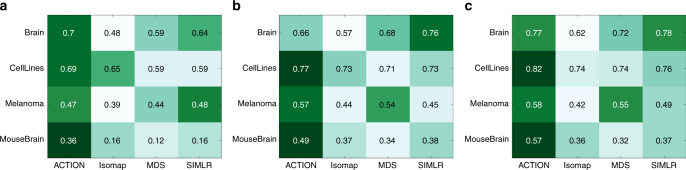
Performance of ACTION Similarity Metric. Various extrinsic measures of clustering quality for different cell similarity scores. **a** Adjusted Rand Index (ARI), **b** F-score, and **c** Normalized Mutual Information (NMI). All of these measures are upper-bounded by one with larger values indicating better results. The results in the table are the mean value over 100 individual runs of kernel k-means clustering with different initializations. The ACTION metric has no tunable parameters. For the other methods, we tested a range of parameters and report the best results. For each dataset, the corresponding row has been color-coded such that the darker green indicates better performance. Except for the Brain dataset, ACTION is either the best, or jointly the best. For Brain, the SIMLR metric is slightly better in an aggregation over all three measures

Figure 3 present the performance of the cell-type identification technique when operating with different similarity measures. Our results demonstrate that in all cases the ACTION metric either outperforms or is jointly the best among competing metrics, except in the Brain dataset in which case SIMLR performs better when looking at all measures combined. A detailed analysis of the underlying distributions and the significance of differences among the top-ranked vs. the runner-up methods is provided in the Supplementary Note 3. Additionally, for the CellLines dataset, which is specifically designed to evaluate cell-type identification methods, we report the heatmap of marker genes for identified cell types to facilitate the visual assessment of the clustering differences, which is also available in Supplementary Note 4.

**Fig. 4:**
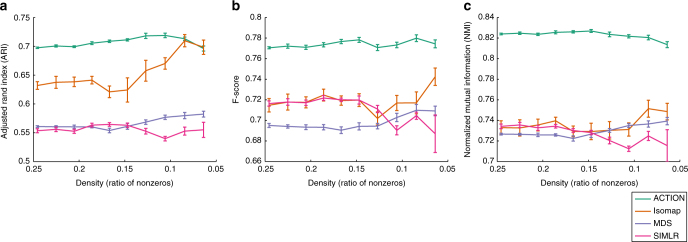
ACTION Kernel Robustness. A series of expression profiles with varying degrees of dropout has been simulated from the CellLines dataset. In each case, we compute different metrics and use kernel k-means to identify cell types. The quality of cell-type identification is assessed with respect to known annotation from the original paper using three different extrinsic measures: **a** Adjusted Rand Index (ARI), **b** F-score, and **c** Normalized Mutual Information (NMI). These results show that ACTION and MDS have the most stable performance over dropout. Error bars correspond to repeated samples of perturbed expression profiles

To assess whether ACTION kernel can extract weak cell-type-specific signals with increasing levels of dropout, we focus on the CellLines dataset that is specifically assayed to evaluate different cell-type identification methods. We created a series of simulated expression profiles, seeded on the CellLines dataset, to mimic different levels of dropout. We iteratively removed nonzero elements at random, with the probability of removal being inversely proportional to the expression value, following previous work^14^. More specifically, the probability of removing each element is (1)/(2^6*x*^), where *x* is the expression value. For each case, we generated 10 independent replicas and used each of them to compute different cell similarity metrics. Finally, we used each metric with kernel k-means and traced changes in the quality of clustering, which is presented in Fig. 4. The ACTION method has the most stable behavior (RSS of the linear fit) with a minor downward trend as density goes below 10%. Furthermore, in each data point, ACTION has lower variation among different replicas. Other methods start to fluctuate unpredictably when density goes below 15%.

Overall, these results establish the ACTION metric as a fast, non-parametric, and accurate method for computing similarity among single cells. We use this measure throughout the rest of our study.

### Uncovering functional identity of single cells

Using the ACTION metric as a measure of similarity between cells, we develop a new method for characterizing the functional identity of cells in a given experiment. Our method is based on a geometric interpretation of cellular functions. In this view, each cell corresponds to a data-point in a high-dimensional space. Our method identifies 'extreme' corners, or archetypes in this space, each of which represents a primary function. The functional identity of each cell is subsequently characterized as a convex combination of these primary functions. (A convex combination is a linear combination of points, such that all coefficients are non-negative and sum to 1.) The choice of the number of primary functions or archetypes is based on a novel non-parametric statistical procedure. See Methods section, Characterizing the functional space, for a detailed description.

To approximate discrete cell types from the primary functions identified using ACTION, we assigned each cell to a single dominant function, as determined by its closest archetype. We compare our method to five recently proposed methods: Seurat (v2.2)^15^, SNNCliq^7^, BackSPIN^16^, single-cell ParTI^8,17^, and TSCAN^9^ (Supplementary Note 1) to predict annotated cell types on the same four datasets (see Methods, Datasets). For the Melanoma dataset, SNNCliq did not terminate after 72 h, after which we stopped the experiment.

**Fig. 5:**
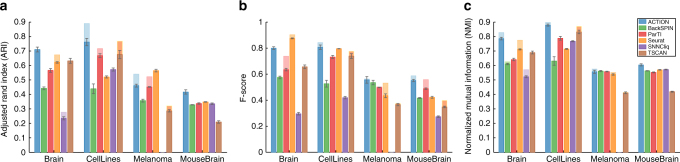
Performance of ACTION in identifying discrete cell types. ACTION identifies cell types by classifying cells according to their dominant primary function (closest archetype). Performance is measured via various measures with respect to the cell types provided with the data: **a** Adjusted Rand Index (ARI), **b** F-score, and **c** Normalized Mutual Information (NMI) of cell-type identification. Larger values are better, and the perfect score (upper bound) is one. Lighter shades are the actual results when using all cells/ samples, whereas the darker bar and the error bar indicates the standard error in a 10-fold test to estimate the variability and stability of predictions for each method. In the CellLines dataset, which was originally created to benchmark cell-type identification methods, ACTION outperforms other methods with respect to ARI and NMI measures, and ties with Seurat in terms of F-score. In the MouseBrain dataset, ACTION significantly outperforms other methods in all three measures. In the Brain datasets there is a competition between ACTION and Seurat, whereas in the Melanoma there is more variability among different methods. This is particularly associated with the level of annotations in this dataset (lack of annotations for T-cell subclasses and tumor subtypes, for example) and the varying resolution of different methods

We report the results of each method applied to each dataset. In addition, to further validate these results, we select 90% of cells in each dataset, proportional to the total cell-type counts, and run each method on each of these 10-folds, and report mean and standard deviation of these results. In all cases, we observe that ACTION performs as well or better than the other methods (Fig. 5). For the Melanoma dataset, however, there is no consensus among the top-ranked methods. This can be attributed, in part, to the extent of available annotations in this dataset and the varying resolution of different methods. We further investigate our results on this dataset in the following sections.

In terms of computational time, graph-based techniques, such as SNNCliq and Seurat, perform better than ACTION for smaller datasets; however, ACTION scales more gracefully as the size of the dataset increases (see Supplementary Note 8 for the details). Also, an example heatmap for the results of the CellLines dataset is provided in the Supplementary Note 5 for an illustration of the benefits of our approach.

In Supplementary Note 9, we study the robustness of ACTION in presence of noise and outliers, as well as its sensitivity to identify rare cell types. We found that preconditioning the adjusted expression profiles significantly enhances the accuracy of predictions, while relaxing the pure pixel assumption further stabilizes these predictions. Furthermore, we show that our method is sensitive enough to identify rare cell types with 2% of the total population. Below this population, they are characterized as noise and outlier cells.

Overall, these experiments show that ACTION, while designed to explore the continuous functional space of cells, is successful in identifying discrete cell types as stable states in this space.

**Fig. 6:**
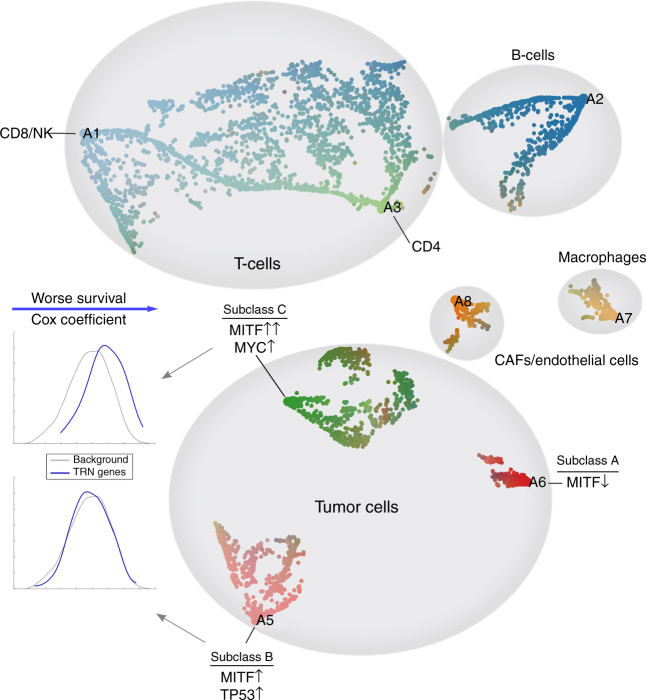
A continuous view on the space of primary functions in the Melanoma dataset. Each archetype, representing a primary function, is illustrated using a textual label (A1–A8). Each small dot represents a cell. Cells are color-coded based on their proximity to archetypes. All data points are projected onto a 2D plane using a carefully initialized Stochastic Neighbor Embedding method (SNE, see Supplementary Note 10). The functional space of cells exhibit a mix of cell state continuum, such as in the case of T-cells, as well as discrete cell types. Three subclasses of melanoma tumor cells are marked accordingly in the map. Subclasses B and C are both MITF-associated. Among them, genes that participate in the transcriptional regulatory network (TRN) for subclass B do not show any significant shift in Cox coefficient, compared to the background of all genes, whereas in subclass C they do. In this sense, high-expression of genes in the TRN of subclass C is significantly associated with worse outcome in the melanoma patients

While the functional identities of cells can be discretized to define cell types, they can also be explored in the continuous space of all primary functions. To illustrate this continuous view, we perform a case study on the Melanoma dataset (Fig. 6). Each point corresponds to a cell. Given the functional profile of cells, defined in a *k*-dimensional space, with *k* being the number of archetypes, we map cells to a two-dimensional plane using the Stochastic Neighbor Embedding (SNE) method with a deterministic initialization (see Supplemental Note 10). Our non-parametric method selected 8 archetypes for the Melanoma data, each is marked with a text label (A1, …, A8) and assigned a unique color. We interpolate the color of each cell using its distance from all archetypes to highlight the continuous nature of the data. We use markers from LM22 dataset^18^ to distinguish different subtypes of T-cells. For the tumor cells, we perform further analysis of active transcription factors, as described in the next section and the methods section, to identify key driving regulators that distinguish each archetype.

Figure 6 demonstrates the ability of our method to identify both isolated cell types with specialized primary functions, as well as the ones with a mixed combination of functions. As an example, T-cells constitute a continuous spectrum across functional space of cells, which is consistent with previous studies^19^. Subclasses of melanoma cells, on the other hand, exhibit distinct separation and have unique phenotypic behaviors and survival rates. In what follows, we identify key marker genes for each subclass, transcription factors that are significantly associated with regulating these genes, and construct their gene regulatory network.

### Constructing cell-type-specific regulatory networks

**Fig. 7:**
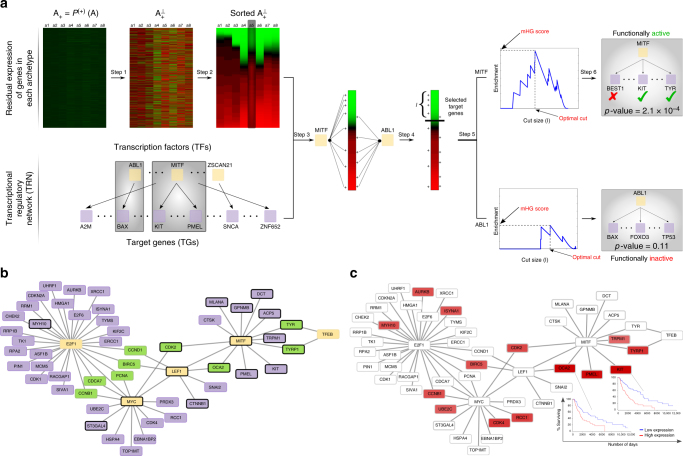
The transcriptional regulatory network (TRN) for MITF-associated Melanoma patients highlights a number of genes that have not previously been associated with Melanoma—along with some known markers. **a** Main steps involved in the construction of archetype-specific TRNs: (1) Orthogonalize archetypes with respect to each other, (2) Sort genes based on their residual expression, (3) Map gene targets for TFs to the sorted list genes, (4) Enrichment analysis for fixed cut size *l*, (5) Find optimal cut size and compute minimum HyperGeometric (mHG) score, and (6) Assess significance of the mHG score using Dynamic Programming (DP). **b** A subset of the TRN of subclass A induced by using only the most significant TFs. The yellow nodes are transcription factors (TF), the purple nodes are target genes (TG), and green nodes are target genes that bridge different TFs. Genes marked with black border are known to be involved in the proliferative subclass of Melanoma. **c** The TRN of subclass A with genes color-coded according to their Cox coefficient. Red genes are the ones whose high expression is associated with worse outcome, and brightness of the color relates to the severity of the outcome. Kaplan–Meier plots for two of the targets of MITF that are unique to subclass A, but not subclass C are shown on the plot

We present a new method for constructing regulatory pathways responsible for mediating the phenotypes associated with each archetype. We first perform an archetype orthogonalization to compute a residual expression and identify marker genes that are unique to each archetype. We then assess the role of each transcription factor (TF) in controlling these marker genes. Significant TFs, together with their top-ranked target genes (TGs), constitute the underlying TRN that is responsible for mediating a given primary function, and consequently, the phenotype associated with cells dominantly associated with that function (see Methods, Constructing the TRN, and Fig. 7a for additional details).

To evaluate the quality of top-ranked genes identified after orthogonalizing each archetype, we selected the top 20 genes and marked the ones that are known markers (according to the original paper) for the cell-type that is enriched for the archetype. Supplementary Note 11 presents a complete table of these top-ranked genes, where known marker genes are in bold typeface. Upon initial observation, a large fraction of these genes appear to be associated with known markers. To systematically assess the significance of this event, we created a label vector for each archetype according to its sorted list of genes after orthogonalization. Then, we use mHG *p*-value to assess the enrichment of markers among top-ranked genes, which are presented in the last row in the table. It is notable that all archetypes are highly significant with respect to the enrichment of marker genes among their top-ranked residual genes, with the exception of CD4 T-cell and tumor subclass A. After further examination, we observed that the majority of T-cell markers provided in the paper are for CD8 T-cell and provided tumor markers in this dataset are for MITF over-expressed melanoma tumors. Thus, the corresponding columns have less significant results that the others.

Next, to distinguish different subclasses of tumor cells, we computed the TFs that are significantly associated with regulating the top-ranked marker genes for each archetype, as well as the particular subset of TGs that they regulate. We found that both subclasses B and C are associated with SOX10 and MITF, two of the most well-characterized markers for 'proliferative' melanoma tumors^20^. Further analysis of these factors, however, reveals that while both of these subclasses are MITF-associated, the degree of association is higher for subclass C. Examining downstream targets of MITF that are activated in each subclass (see Supplementary Note 12), we identified that GPNMB, MLANA, PMEL, and TYR are shared between two subclasses, whereas ACP5, CDK2, CTSK, DCT, KIT, OCA2, and TRPM1/P1 are unique to subclass C. To validate these targets, we used a comprehensive list of downregulated genes in response to MITF knockdown in 501Mel melanoma cells^21^. The overlap of identified MITF TGs and the set of downregulated targets was significant for subclasses B and C (hypergeometric test *p*-values of 7.5 × 10^−5^ and 1 × 10^−6^, respectively). This further validates that our method is identifying not only the right TFs, but also the right set of TGs for them. Among other distinguishing TFs, subclass B is significantly associated with BRCA1 and TP53, whereas subclass C is associated with MYC. Factors BRCA1 and TP53 are both tumor-suppressors, whereas MYC is a proto-oncogene. Activation of these transcriptional factors, in turn, can differentially regulate downstream targets that may contribute to worse outcome in subclass C.

Based on these observations, we propose the hypothesis that subclass C should have worse outcome than subclass B. To support this hypothesis, we construct subclass-specific TRN for these two subclasses. The set of TFs in these networks have a total of 51 and 91 distinct TGs, respectively, that are functionally active. In order to understand how the difference among these genes contribute to the overall survival of patients, we assessed the association between identified genes in each network and survival rate of Melanoma patients in the TCGA dataset, measured via multivariate Cox regressions^22^. We note that genes in subclass C significantly deviate from the null distribution of Cox coefficients for all genes (Kolmogorov–Smirnov test; *p*-value = 5.4 × 10^−10^), whereas genes in subclass B do not (*p*-value = 0.31), which translates into worse prognosis for subclass C. These observations are summarized in Fig. 6.

To further study the underlying regulatory mechanisms that drive this poor-outcome phenotype for subclass C, we focus on only the most significant TFs (those with functional activity *p*-values ≤10^−3^, rather than ≤0.05 above) and construct their associated regulatory network. Figure 7a shows the interaction network among highly significant TFs and their major targets in subclass C. While some of these factors, and their TGs, were previously directly or implicitly associated with Melanoma, this network provides a novel systems view of the interactions, and highlights new regulatory interactions. For instance, amplification of the MYC oncogene has been long associated with poor-outcome in Melanoma patients^23^. Also, E2F1 is a critical TF that is involved in cell cycle transition from G1 to S phase, and its overexpression is commonly associated with poor patient survival in high-grade tumors^24^. The LEF1 factor has a dual role. On one hand, it acts as a downstream effector of the Wnt signaling pathway and is associated with phenotype switching in Melanoma cells between proliferative and invasive states^25^. On the other hand, it has been suggested that LEF1 has a distinct, Wnt-independent, role in activating E2F1^26^. Finally, we note that LEF1 regulates both MITF and MYC. Collectively, we hypothesize that LEF1 is a key TF that regulates phenotype switching from proliferative to invasive state in subclass C, by controlling other TFs, including MITF, MYC, and E2F1.

To revisit the problem of survival analysis, and to recover genes that affect this prognostic change, we project individual Cox coefficients for each gene onto the TRN of subclass C (Fig. 7b). Two of the most significantly associated genes, *KIT* and *OCA2*, are among *MITF* targets that are unique to subclass C, but not subclass B. The Kaplan–Meier plots for these two genes are visualized alongside the TRN. In addition, there are multiple targets of MYC, LEF1, and E2F1 that are also associated with poor outcomes for melanoma patients.

Finally, to assess the therapeutic indications of these subclasses, we used the pharmacogenomic profiling of cancer cell lines^27^. There are 53 melanoma cell lines in this dataset. For each of these cell lines, we have access to both their transcriptomic profile and drug response for 256 different drugs. We used the top 100 genes in subclasses A–C to find cell lines that closely resemble each of these subclasses. We *z*-score normalize each row of this submatrix and use mHG *p*-value to assess the the enrichment of marker genes among top-ranked genes. We use a *p*-value of 10^−3^ to ensure that selected cell lines are closely related to original subclasses. This leaves us with 9, 6, and 15 cell lines for subclasses A, B, and C, respectively, and 23 unclassified cell lines. For cell lines associated with subclasses B and C, we used a *t*-test to assess differences in the distribution of IC50 value between these two subclasses. We found that subclass C is more sensitive to the drugs targeting ERK MAPK signaling, specifically Refametinib, CI-1040, PLX-4720, SB590885, Selumetinib, AZD6482, PLX-4720, and Dabrafenib, among which PLX-4720 and Dabrafenib are the most effective ones.

## Discussion

We present a unified framework for characterizing the state space of single-cell transcriptomes. Our approach, motivated by archetypal analysis (AA), identifies dominant landmarks, or archetypes, among the population of cells. All other cells are then represented with respect to their relationship to these archetypes. This, in turn, simplifies the interpretation of our results, since each archetype corresponds to the transcriptional signature of a putative cell type. By using a locally sparse averaging scheme, our framework alleviates the problem of dropouts, which is a fundamental challenge in the single-cell analysis, while preserving as much of the cell state information as possible. Put together, ACTION provides a natural decomposition that is easy to interpret, facilitates marker detection, and can be applied to both characterize the continuous state of cells, as well as discrete cell types.

## Methods

### Datasets

Single-cell gene expression datasets: For all our studies, we rely on the following datasets collected from publicly available sources:

Brain (GEO: GSE67835): This dataset contains 466 cells spanning various cell types in the human brain, including astrocytes, oligodendrocytes, oligodendrocyte precursor cells (OPCs), neurons, microglia, and vascular cells^28^.

CellLines (GEO: GSE81861): This dataset is recently published to benchmark existing cell-type identification methods. It contains 561 cells from seven different cell lines, including A549 (lung epithelial), GM12878 (B-lymphocyte), H1 (embryonic stem cell), H1437 (lung), HCT116 (colon), IMR90 (lung fibroblast), and K562 (lymphoblast). To assess the effect of batch effects, GM12878 and H1 are assayed in two batches^29^.

Melanoma (GEO: GSE72056): This dataset measures the expression profile of 4645 malignant, immune, and stromal cells isolated from 19 freshly procured human melanoma tumors. These cells are classified into 7 major types^12^.

MouseBrain (GEO: GSE60361): This dataset contains the expression profile of 3005 cells from the mouse cortex and hippocampus. These cells classify into seven major types, including astrocytes-ependymal, endothelial-mural, interneurons, microglia, oligodendrocytes, pyramidal CA1, and pyramidal SS^16^.

Transcriptional Regulatory Network (TRN): We collect TF–TG interactions from the TRRUST database^30^. This dataset contains a total of 6314 regulatory interactions between 651 TFs and 2102 TGs.

Drug sensitivity in cell lines: We downloaded processed gene expression and drug sensitivity data from the Genomics of Drug Sensitivity in Cancer Project website^27^. These datasets consist of a total of 1001 cell lines, spanning different types of cancer, 52 of which are melanoma cell lines that also have their gene expression profile available. A total of 256 compounds were screened on these cell lines IC59 values for each pair has been reported.

### Computing ACTION metric as a measure of similarity between cells

The transcriptome of each cell consists of genes that are expressed at different levels and have different specificity with respect to the underlying cell types. Universally expressed genes correspond to the subset of genes responsible for mediating core cellular functions. These functions are needed by all cells to function properly, which result in ubiquitous expression of these genes across all cell types^31^. While fundamental to cellular function, these genes are not informative with respect to the identity of cells. On the other hand, cell-type-specific genes are preferentially expressed in one or a few selected cell types to perform cell-type-specific functions. Unlike universally expressed genes, cell-type-specific genes are, typically, weakly expressed, but are highly relevant for grouping cells according to their common functions. Our goal here is to define a similarity measure between cells that suppresses universally expressed genes and enhances the signal contained in cell-type-specific genes.

To suppress the ubiquitously high expression of universally expressed genes, we adopt a method that we developed recently for bulk tissue measurements and extend it to single-cell analysis^10^. This method projects a standardized representation of expression profiles of cells onto the orthogonal subspace of universally expressed genes. Let us denote the raw expression profile of cells using matrix ${\mathbf{X}} \in {\Bbb R}^{m \times n}$, where each row corresponds to a gene and each column represents a cell. We use ***x***_*i*_ to denote the expression profile of *i*^*th*^ cell. In addition, let us denote the signature vector of universally expressed genes by ***v***. As a first order estimate, a universally expressed signature is computed by taking the average expression over all cells: ${\mathbf{v}} = \frac{1}{n}\mathop {\sum}\nolimits_{i = 1}^n {\mathbf{x}}_i$; that is, ***v***_*i*_ is the average expression of gene *i* across all samples. This choice is motivated by the fact that highly expressed genes are more consistently expressed, whereas lowly expressed genes show exhibit higher variability. To this end, by orthogonalizing with respect to the mean value, we significantly reduce the effect of universally expressed genes, while preserving the variation of lowly expressed, but preferential ones^32^. After estimating this baseline expression, we *z*-score normalize the profile of each cell: ${\mathbf{z}}_i = \frac{{{\mathbf{x}}_i - \mu _i}}{{\sigma _i}}$, where *μ*_*i*_ and *σ*_*i*_ are the mean and sample standard deviation of the entries in the *i*th cell profile. Similarly, we *z*-score normalize the signature vector of universally expressed genes, ***v***, to create a new vector ***z***_*v*_. Finally, we project out the impact of the universally expressed gene expressions on each cell’s profile as follows:

1 $${\mathbf{z}}_i^ \bot = ({\mathbf{I}} - \frac{{{\mathbf{z}}_v{\mathbf{z}}_v^T}}{{\left| {\left| {{\mathbf{z}}_v} \right|} \right|_2^2}}){\mathbf{z}}_i.$$

This operation projects ***z***_*i*_ to the orthogonal complement of the space spanned by the universally expressed genes. We then concatenate the column vectors ${\mathbf{z}}_i^ \bot$ to create a adjusted cell signature matrix ${\cal Z}^ \bot$.

Next, to enhance the signal contributed by preferentially expressed genes, we propose an information theoretic approach that is inspired by the work of Schug et al.^33^. The main idea is to use Shannon’s entropy to measure the informativeness of genes. If a gene is uniformly utilized across cells, it contains less information as opposed to the case in which it is selectively active in a few cells. To this end, we start with the positive projection of adjusted cell signatures, ${\cal P}^{( + )}\left( {{\cal Z}^ \bot } \right)$, in which case we replace all negative values with zeros. Then, we normalize this matrix to construct a stochastic matrix ***P*** (every row sums to one). Let ***p***_*i*_ be the row vector associated with the *i*^*th*^ gene. We compute the uniformity, or normalized entropy, of ***p***_*i*_ as: *u*(*i*) = −∑_*j*_*p*_*ij*_log(*p*_*ij*_)/log(*n*), where *p*_*ij*_ is an entry in the matrix **P** and *n* is the number of genes. This value is always between zero and one and is used as a basis to boost contributions from the most informative genes. A detailed comparison of our entropy-based method with dispersion and Gini index is provided in the Supplementary Note 2.

To scale genes according to their specificity, we compute a coefficient that controls the contribution of each gene. This coefficient is greater than one (scales up) for cell-type-specific genes and less than one (scales down) for universally expressed genes, respectively. To do so, we note that the distribution of the entropy values follows a bimodal distribution, with separate peaks for the cell-type-specific and universally expressed genes. To identify the critical point where these two population separate from each other, we fit a mixture of two Gaussians over the distribution of the values and use it to identify this transition point, denoted by $\hat u$, which is the point of equal probability from each Gaussian.Then for each gene *i*, we define a scaling factor as $w_i = \hat u{\mathrm{/}}u(i)$. Finally, we compute the kernel matrix as follows:

2 $${\mathbf{K}}{\mathrm{ = }}({\cal Z}^ \bot )^T\mathrm{diag}({\it{w}}^2){\cal Z}^ \bot$$

In this formulation, if we denote ${\bf{Y}}={\mathrm{diag}}\left(w \right){\cal Z}^ \bot$, then **K** is a dot-product kernel defined as **Y**^*T*^**Y**. We will refer to **Y** as the adjusted transcriptional profile of cells, and **K** as the cell similarity kernel, or ACTION metric.

### Characterizing the functional space of individual cells

Due to evolutionary constraints, biological systems, including cells, that need to perform multiple primary functions, or tasks, can not be optimal in all those tasks; thus, these systems evolve to be specialized in specific tasks^11^. The functional space of cells then can be represented by a low-dimensional geometric construct, such as a polytope, the corners of which correspond to the set of specialized primary functions. The convex hull of a given set of points is the minimum volume polytope that encloses all points. This can be envisioned as a rubber band fitting to the outermost points. Constructing the convex hull in high-dimensional space is computationally expensive and susceptible to noise and overfitting. As an alternative, we seek a limited number of points on the convex hull that enclose as many points as possible, while being resilient to noise and outliers. Each point here represents a cell and each corner, or archetype, of this polytope is a candidate cell that best represents a unique primary function. To find these candidate cells, we use a modified version of the successive projection algorithm (SPA) combined with a novel model selection technique to identify an optimal number, *k*, of candidate cells on the approximate convex hull that best represent distinct pure cells with specialized primary functions. Finally, we use the principal convex hull algorithm (PCHA) to relax these corners to allow others cells to contribute to the identity of each archetype/corner.

Formally, given a matrix **Y** representing the adjusted transcriptional profile of cells, we aim to construct an optimal set ${\cal S}$ of *k* columns such that each selected column is an ideal representative of the cells that perform a given primary function. Let us assume that matrix **Y** can be decomposed as ${\mathbf{Y}} = {\mathbf{Y}}(:,{\cal S}){\mathbf{H}} + {\mathbf{N}}$, where ${\cal S}$ is the selected column subspace of matrix **Y**, **H** is non-negative with column-sums equal to one, and **N** represents bounded noise, where $\parallel {{\mathbf{N}}(:,j)} \parallel_2 \le \varepsilon$. That is, we can select $|{\cal S}| = k$ columns from matrix **Y** to represent rest of the columns, with consideration for noise. A matrix satisfying this condition is called near-separable and is known as the near-separable non-negative matrix factorization (NMF) when **Y** is non-negative. For a matrix satisfying near-separability, there is an efficient algorithm, with provable performance guarantees, that can identify columns in ${\cal S}$. Furthermore, premultiplying matrix **Y** with a nonsingular matrix **Q** preserves its separability, but if chosen carefully, can enhance the conditioning of the problem and accuracy of results. To find the optimal preconditioning matrix **Q**, we use a theoretically grounded method based on identifying a minimum volume ellipsoid at the origin that contains all columns of **Y** (Supplementary Note 6).

Given that SPA selects *k* columns of **Y**, *given k*, the next issue is how to find the optimal value of *k* that captures most variation in data without overfitting. We devised a novel monitoring technique that assesses the current *k*-polytope to see if there is any evidence of oversampling the cell-space. If so, it stops the algorithm. Otherwise, it continues by adding new archetypes. Informally, oversampling happens when we start adding new archetypes to regions in the space that are already well-covered by other archetypes, in which case the newly added archetype would be significantly close to one or more other archetypes, compared to the rest of the archetypes. Given that each archetype is a candidate cell, we can measure relationship between them using the ACTION metric. The distribution of similarities resembles a normal distribution; however, as we start to oversample, the right tail of the distribution starts getting heavier. To distinguish the pairs of archetypes in this heavy-tailed region, we *z*-score normalize pairwise similarities between archetypes and select all pairs whose *z*-transformed similarity scores are above 1.96, which corresponds to 95% confidence level under Gaussian assumption for the underlying distribution. Then, we build an archetype similarity graph using these pairs of close archetypes. In this graph, oversampling can be identified by the emergence of dense local regions. We use the Erdös-Rényi (ER) random graph model as a background to assess density of each sub-region, or connected component, in the archetype similarity graph^34^. If we find at least one of the connected components that is significantly dense, which is a sign of oversampling, then we terminate the algorithm and choose the last value of *k* before oversampling happens.

After estimating *k* ideal candidate cells, or pure cells, we use AA^35^, which can be viewed as a generalization of near-separability to relax corners by locally adjusting them to have contributions from multiple cells. Formally, we can formulate AA as follows:

3 $$\begin{array}{*{20}{l}} {\begin{array}{*{20}{c}} {{\mathrm{minimize}}} \\ {{\mathbf{C}},{\mathbf{H}},{\mathbf{\alpha }}} \end{array}} \hfill & {\parallel {{\mathbf{Y}} - {\mathbf{YCH}}} \parallel} \hfill \\ {{\mathrm{subject}}\,{\mathrm{to}}} \hfill & {\parallel {{\mathbf{C}}(:,i)_1 = 1} \parallel.} \hfill \\ {} \hfill & {\parallel {{\mathbf{H}}(:,i)_1 = 1} \parallel.} \hfill \\ {} \hfill & {0 \le {\mathbf{C}},0 \le {\mathbf{H}}} \hfill \end{array}$$

Near-separable non-negative matrix factorization is a special case of AA in which **Y** is non-negative, **C** has exactly *k* nonzeros, and none of the columns have more than one element. We use an efficient algorithm, called Principal Convex Hull Analysis (PCHA), to solve the above problem to a local optima.

The matrix **A** = **YC** then stores the archetypes. Column stochasticity of **C** indicates that archetypes are convex combinations of data points, and column stochasticity of **H** indicates each data point can be represented as convex combination of archetypes.

A complete pseudo-code fitting all these components together is provided in Supplementary Note 7.

### Constructing the TRN for each archetype

In order to understand what control mechanisms are responsible for mediating the transcriptional phenotype of each archetype, we first have to identify key marker genes that distinguish a given archetype from the rest of archetypes (see Fig. 7a for an illustrative guide to this section). To this end, we first orthogonalize each archetype with respect to all other archetypes. In this formulation, what remains, referred to as the residual expression of genes, ranks genes according to their importance in a given archetype. Let matrix **A** = **YC** represent the identified archetypes. Let ${\mathbf{A}}^{( + )} = {\cal P}^{( + )}({\mathbf{A}})$ be the projection to positive entries and let ${\mathbf{a}}_i^{( + )}$ stand for the column *i* of **A**^(+)^. Moreover, let ${\mathbf{A}}_{ - i}^{( + )}$ denote the matrix without the *i*th column. Our goal is to project ${\mathbf{a}}_i^{( + )}$ into the subspace orthogonal to the columns spanned by ${\mathbf{A}}_{ - i}^{( + )}$. Then, the orthogonalization step can be written as:

4 $${\mathbf{a}}_i^ \bot {\mathrm{ = }}\left( {{\mathbf{I}}{\mathrm{ - }}{\mathbf{A}}_{ - i}^{( + )}\left( {{\mathbf{A}}_{ - i}^{( + )^T}{\mathbf{A}}_{ - i}^{( + )}} \right)^{ - 1}{\mathbf{A}}_{ - i}^{( + )^T}} \right){\mathbf{a}}_i^{( + )}$$

Finally, we construct matrix ${\mathbf{A}}_ + ^ \bot$ where each column is ${\mathbf{a}}_i^ \bot$. Terms in this matrix are called residual expressions and help identify distinguishing marker genes for each archetype.

Those genes with high residual expression in each archetype are controlled through regulatory networks within the cell. To uncover these relationships, we identify TFs that are significantly associated with the expression of marker genes, which we will refer to as functionally active TFs. Functional activity of TFs is inferred directly from the expression of their TGs; thus, these TF activities can be controlled at different stages, ranging from transcriptional to post-translation regulations. To infer these activities, we first need to classify their TGs as either active or inactive in a given context (archetype). We partition genes according to their residual expression and declare top-ranked genes as active. We use the minimum hypergeometric (mHG) method^36^ to find the optimal partition of genes and assign a *p*-value to it. The main step of this algorithm is similar to classic enrichment analysis: for a fixed size *l*, we use the hypergeometric *p*-value to assess the over-representation of TGs for a given TF among top-*l* markers for an archetype. Then, we compute the same statistic for all 1 ≤ *l* ≤ *m*, where *m* is the total number of genes. The mHG tail that is obtained, referred to as the mHG score, specifies the best cut, *l*^(*best*)^, and all TGs that are ranked higher than *l*^(*best*)^ among marker genes are selected as regulated targets for that TF. Finally, we use the obtained mHG score to assess the significance of the TF itself. This can be accomplished using a dynamic programming algorithm that assesses the probability of observing the same or more significant mHG score within the population of all binary vectors of size *m* with exactly *r* nonzeros, where *r* is the number of targets for the current TF. The set of all significant TFs, together with their TGs that fall above the cut that results in the mHG score, are used to construct the final TRN.

### Code availability

All codes are available in C/C++ with R/Matlab interfaces from http://compbio.mit.edu/ACTION.

### Data availability

The data that support the findings of this study are available from the corresponding author upon reasonable request.

## Electronic supplementary material

Supplementary Information
