# Supplementary material for: A geometric approach to characterize the functional identity of single cells
Source: Nat Commun. 2018 Apr 17;9:1516. doi: 10.1038/s41467-018-03933-2 (PMC5904143; doi:10.1038/s41467-018-03933-2)
Supplement: Supplementary file 1 — Supplementary Information [file 41467_2018_3933_MOESM1_ESM.pdf]

Supplementary Information

Supplementary Note 1: Overview of prior methods for cell-type identification

Various methods have been developed for cell type identification. **SNNCliq** [1] computes a similarity graph among cells, referred to as *shared nearest neighbor (SNN)*. It then uses a graph-based clustering algorithm to identify dense subgraphs. **Seurat** [2] was originally designed for spatial reconstruction of scRNA-Seq data. Since then, it has been extensively updated and used for cell-type identification. In more recent versions (v2.2), Seurat adopted a graph-based approach similar to SNNCliq with extensive modifications that deviate from the original version. **TSCAN** [3] starts by grouping genes with similar expression patterns into "modules" and represents all cells in this reduced space. It then performs principal component analysis (PCA) over the module space to further reduce dimensions. Finally, cells are clustered by fitting a mixture of multivariate normal distributions to the data, with the number of components estimated using the Bayesian Information Criterion (BIC). **SCUBA** [4] first uses k-means with gap statistic to cluster data along an initial binary tree by analyzing bifurcation events for time-course data. Then, it refines the tree using a maximum likelihood scheme. **BackSPIN** [5] is based on the SPIN algorithm, which permutes correlation matrix of cell types to extract its underlying structure. BackSPIN then couples it with a divisive splitting procedure to identify clusters from the ordered similarity matrix. Two methods are specifically designed to identify rare cell types. RaceID [6] uses k-means to first cluster cells, with the number of clusters identified using gap statistic. Then, it identifies rare cell types as outliers that are not explained by an appropriate noise model, accounting for both biological and technical variations. **GiniClust** [7] aims to identify marker genes that are specific to rare cell types using the concept of Gini index. Then, it computes distances between cell

types in this reduced subspace and uses DBSCAN clustering algorithm to identify cell types. In addition to these methods, there are approaches that visualize cell types on a continuous spectrum in a given space. Haghverdi *et al.* [8] use diffusion maps to model the continuous spectrum of cells. In another direction, Korem *et al.* [9], adopted a previously developed method, called **Pareto task inference (ParTI)** [10] and applied it to single cell datasets. The latter method itself is based on the original work of Shoval *et al.* [11]. While ParTI uses a similar notion non-convex archetypal analysis as what we do, our method begins with the separable NMF method, the solution of which can be formulated as a convex problem, to pick "ideal" candidate cells as archetypes. Then, it uses the non-convex PCHA procedure to refine these primary archetypes by sparse local averaging to combat noise in the data. Furthermore, our method is founded on a biologically-inspired, kernel-based approach, has a novel method to identify the number of cell types, and last but not the least, a statistical method to construct regulatory circuits that uniquely distinguish each cell type.

Supplementary Note 2: Comparison of Entropy-based marker detection method with Gini index and dispersion

In order to compare the performance of different methods to identify cell type-specific genes, we focused on the **Melanoma** and the **MouseBrain** datasets, for which the original paper provided curated markers for cell types. For each dataset, we ranked genes according to each measure, both before and after adjustment for the effect of universally-expressed genes. Then, for each cell type, we created a true-positive vector based on its curated markers and assessed the over-representation of these markers among top-ranked genes from each method. Finally, we combined each of these over-representation *p*-values using Fisher's method. Supplementary Figure 1 illustrates the results for each dataset. As can be seen from the figure, in both datasets, the *dispersion* method is superior before adjustment, whereas Gini index and Entropy-based methods excel after adjustment. As a general trend, we observe that dispersion methods outperform in predicting markers for the most frequent cells in the dataset (T and tumor cells in the **Melanoma** dataset, and S1Pyramidal, CA1Pyramidal, and Oligodendrocyte in the **MouseBrain** datasets), whereas the other two methods significantly outperform dispersion for the rest of cell types, including rare cell types.

Next, to evaluate the extent of overlap among top-ranked genes, we focused on the top 1,000 genes in each method. Supplementary Figure 2 shows the Venn diagram for the overlap of datasets. The Gini index and entropy based methods have the highest agreement with each other, while the entropy-based method has a higher overlap with dispersion method than Gini index.

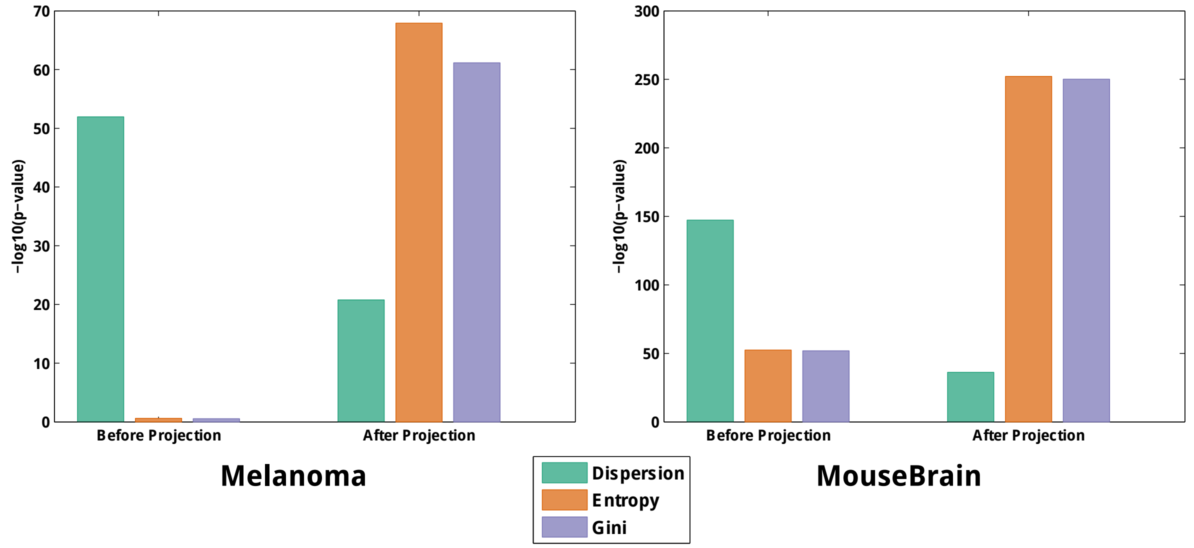

Supplementary Figure 1: Performance of different marker detection methods before/after correction for the effect of universally expressed genes.

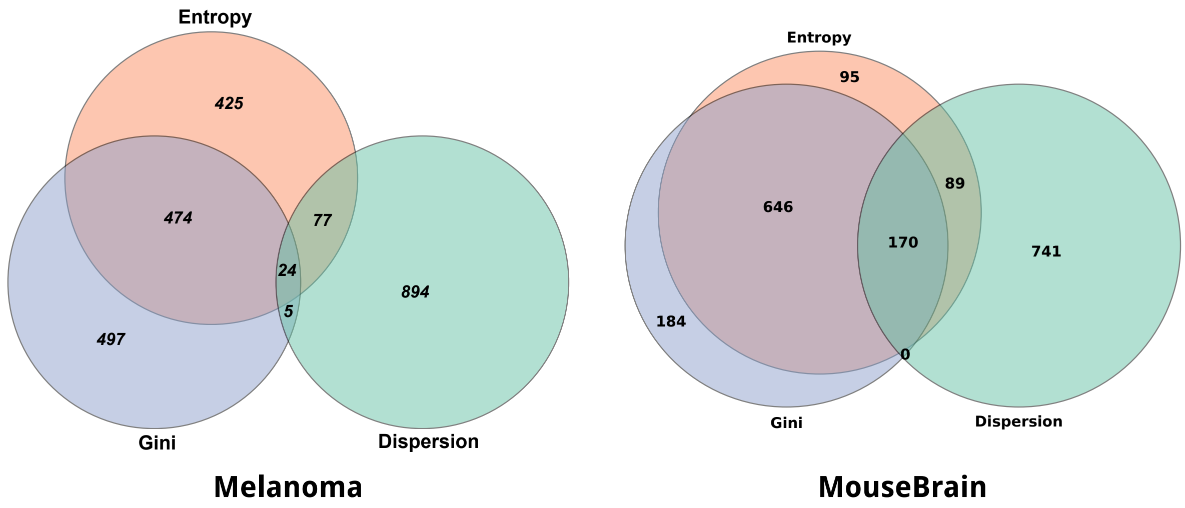

Supplementary Figure 2: Overlap among top-ranked 1,000 genes predicted using dispersion, Gini index, and entropy-based methods.

Supplementary Note 3: Distribution of clustering measures and significance of differences between different cell similarity metrics

In the main text, we reported a mean over 100 trials of kernel k-means with the four kernels: ACTION, IsoMap, MDS, and SIMLR. Supplementary Figure 3 shows the actual distribution of different quality measures for each kernel k-means run. The figure also reports a t-test between the first and second-best method. As in the main figure, ACTION performs equally well or better than other metrics, with the only exception being F-score for the **Brain** dataset.

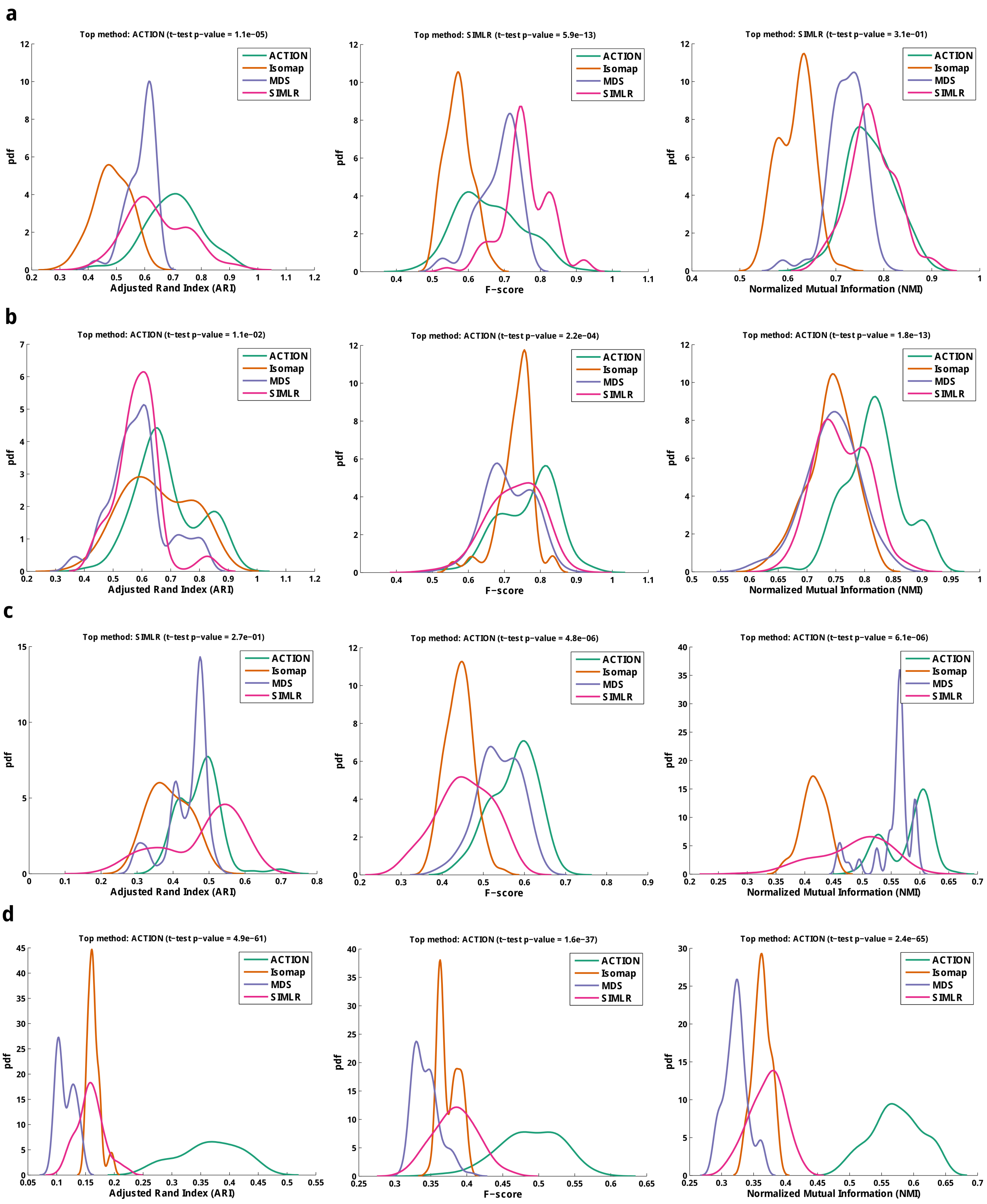

Supplementary Figure 3: **Performance of cell similarity metrics**

Supplementary Figure 3 *(previous page)*: For each extrinsic measure on each dataset, the distribution of values for kernel k-means runs is presented. In each case, the p-value of t-test between the top-ranked versus runner-up methods has been reported. (**a**) **Brain** dataset, (**b**) **CellLines** dataset, (**c**) **Melanoma** dataset, (**d**) **MouseBrain** dataset.

Supplementary Note 4: Detailed analysis of cell types identified using different similarity metrics – case study in the CellLines dataset

The CellLines data contains measurements from seven distinct cell-lines: A549, GM12878, H1, H1437, HCT116, IMR90, K562. We used this dataset to assess the results from kernel k-means for all of the different metrics. The goal was to use a standard algorithm and compare the results as we vary the type of cell-similarity. Supplementary Figure 4 shows the subspace of cell line-specific markers, sorted according to the identified cell types in different methods. In all cases, there exists a predicted cell type that mistakenly mixes samples from the *H1437* cell line with one or more other cell lines. In case of *ACTION*, it almost identifies *H1437* perfectly, with marginal contamination from from the *K562* and *IM90* cell lines. This, however, is not surprising since all three of these cell lines are based on lung tissue.

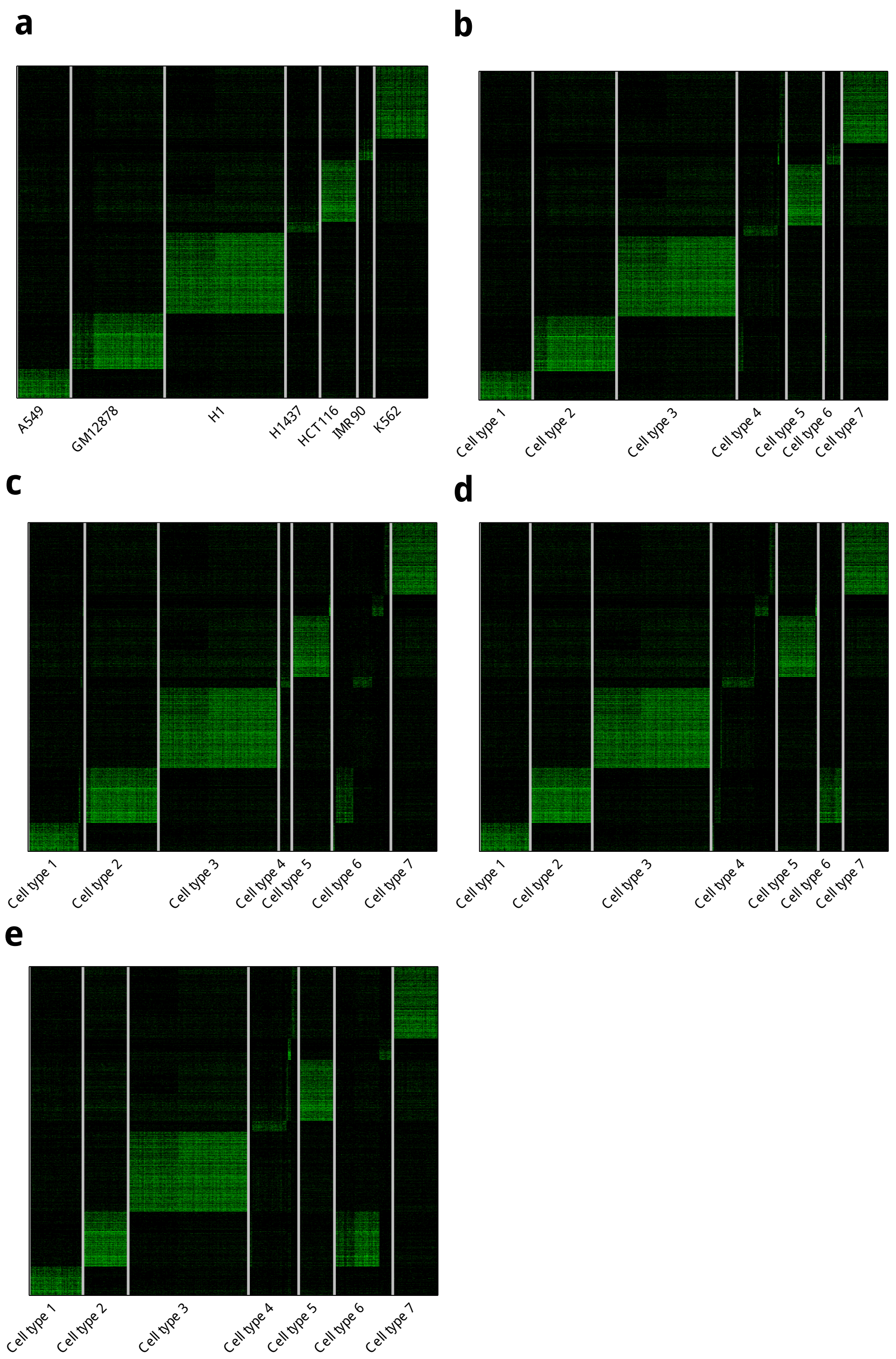

Supplementary Figure 4: **Heatmap of predicted cell types using kernel k-means with different similarity metrics** (**a**) original, (**a**) Original, (**b**) ACTION, (**c**) IsoMap, (**d**) MDS, (**e**) SIMLR

Supplementary Note 5: Detailed analysis of cell types identified using different cell type identification methods – case study in the CellLines dataset

Our next study is similar to the previous one (Supplemental Note). In this study, the goal is to compare cell-type identification methods rather than similarity metrics. (Supplementary Figure 5 shows the marker subspace of identified cell types based on different different cell type identification methods, all of which are nonparametric methods (in the sense that they automatically estimate the number of cell types). Among these methods, *ACTION* that has the highest score and identifies almost all cell types correctly, except that it mixes IMR90 with one of the batches of GM128787. In BackSPIN, all cell lines are either split between two predicted cell types or are mixed with each other. This situation is somewhat better for ParTI, for which the first batch of GM128787 and the H1 cell lines are predicted correctly. However, all other predicted cell types are a mix of different cell lines. SNNCliq splits the cells into too many types. Finally, TSCAN, which performs the second best, mixes parts of K562 and GM128787, and also splits H1 between separate predicted classes. Overall, ACTION shows the highest consistency with the true annotation of cell types, followed by TSCAN.

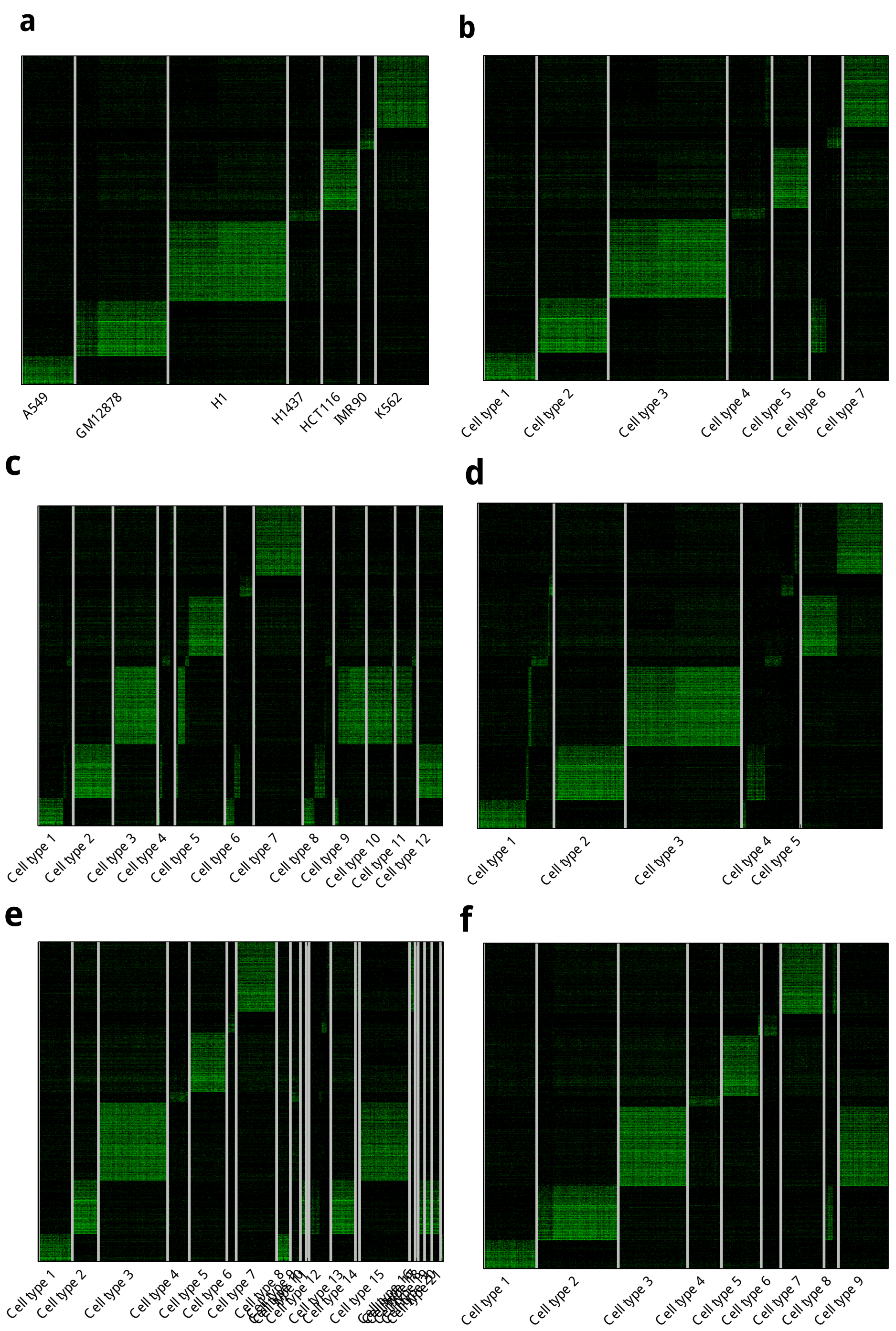

Supplementary Figure 5: **Heatmap of predicted cell types using different cell type identification methods applied to the CellLines dataset** (**a**) original, (**a**) Original, (**b**) ACTION, (**c**) BackSPIN, (**d**) ParTI, (**e**) SNNCliq, (**f**) TSCAN

Supplementary Note 6: Performance of SPA with preconditioner

Let $\mathbf{Y} = \mathbf{WH}$, where matrix $\mathbf{W}$ is defined as $\mathbf{Y}(:, \mathcal{S})$, with $\mathcal{S}$ being the selected column subspace of matrix $\mathbf{Y}$, and $\mathbf{H}$ is a non-negative matrix with column-sums equal to one. Moreover, let matrix $\tilde{\mathbf{Y}} = \mathbf{Y} + \mathbf{N}$, where the noise is bounded: $\|\mathbf{N}(:, j)\|_2 \leq \varepsilon$. Then, the performance of the SPA algorithm has the following upper bound guarantee:

$$\max_{1\leq j\leq k} \min_{s\in\mathcal{S}} \| \tilde{\mathbf{Y}}(:, s) - \mathbf{W}(:, j) \| \leq \mathcal{O}\Big(\epsilon\kappa^2(\mathbf{W})\Big) \tag{1}$$

More recently, other techniques have been developed to enhance the robustness of *SPA* to noise [12]. These methods are based on the fact that premultiplying matrix $\mathbf{Y}$ by a nonsingular matrix $\mathbf{Q}$ preserves its separability. In this case, the upper bound limit changes to: $\mathcal{O}\Big(\epsilon\kappa(\mathbf{W})\kappa^3(\mathbf{QW})\Big)$. Thus, by carefully choosing matrix $\mathbf{Q}$, we can enhance the conditioning of the problem. Ideally, if $\mathbf{Q} = \mathbf{W}^{-1}$, then $\kappa^3(\mathbf{QW}) = 1$ and we reduced the upper bound from quadratic to linear. While $\mathbf{W}^{-1}$ is not accessible, we can approximate $\mathbf{W}^{-1}$ using a *minimum volume ellipsoid* centered around the origin that contains all columns of the original matrix $\mathbf{X}$. Formally, this can be solved using the following SDP to identify matrix $\mathbf{A}^*$:

$$\mathbf{A}^{(*)} = \underset{\mathbf{A}\in\mathbb{S}^k_+}{\operatorname{argmax}}\, \mathbf{det}(\mathbf{A})$$

$$\text{s.t.: } \mathbf{Y}(:, j)^T \mathbf{A} \mathbf{Y}(:, j) \leq 1; \forall j$$

Since $\mathbf{A^T}$ is symmetric positive definite, we compute $\mathbf{A^T} = \mathbf{Q}^T\mathbf{Q}$ using Cholesky factorization and use it as a preconditioner.

Supplementary Note 7: Pseudo-code for fitting a geometric construct over single cells

Algorithm 1 SPA algorithm with prewhitening

Input: $\mathbf{Y} \in \mathbb{R}^{m \times n}$: adjusted expression profile of cells

Output: $\mathbf{A} \in \mathbb{R}^{m \times k}$: primary functions, $\mathbf{H} \in \mathbb{R}_{+}^{k \times n}$: functional identity of cells

Solve **minimum volume ellipsoid** problem to identify preconditioner $\mathbf{Q}$.

$\mathbf{K} = \mathbf{Y}^T\mathbf{Y}, \mathbf{R} = \mathbf{QY}, \mathcal{S} = \{\}$

for $i = \{1, \cdots, max_k\}$ **do**

  $\alpha = \operatorname{argmax}_j \|\boldsymbol{r}_j\|_2$ {$\boldsymbol{r}_j$ is the jth column}

  $\boldsymbol{\beta} = \mathbf{R}(:, \alpha)$

  $\mathbf{R} \leftarrow (\mathbf{I} - \frac{\boldsymbol{\beta}\boldsymbol{\beta}^T}{\boldsymbol{\beta}^T\boldsymbol{\beta}})\mathbf{R}$ {Orthogonal Projection}

  $\mathcal{S} \leftarrow \mathcal{S} \cup \{\boldsymbol{\beta}\}$

  Construct archetype similarity graph from $\mathbf{G} = \mathbf{K}(\mathcal{S}, \mathcal{S})$

  if $subgraph_{density}(\mathbf{G})$ is significant **then**

    break

  end if

end for

Initialize $\mathbf{C}_0$ using selected columns in $\mathcal{S}$, and run kernel **PCHA** with $\mathbf{K}$ to estimate matrices $\mathbf{C}$ and $\mathbf{H}$

$\mathbf{A} = \mathbf{YC}$

Supplementary Note 8: Computational runtime analysis

In terms of timing, the most time-consuming part of *ACTION* is the preconditioning using minimum volume ellipsoid method, which depends on the solver being used. Using CVX with Mosek solver, timings are as reported in Supplementary Figure 6. For larger datasets, it can be seen that *ACTION* scales more gracefully compared to other methods.

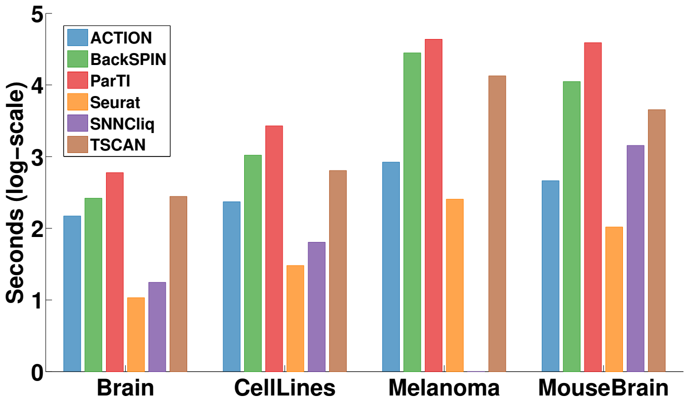

Supplementary Figure 6: Time-wise, smaller values are the better. It can be seen that *ACTION* scales better as the datasets grow.

Supplementary Note 9: Robustness of *ACTION* method in presence of noise and outliers

To further evaluate the effect of preconditioning convex Non-negative Matrix Factorization (NMF), as well as relaxing it with Principal Convex Hull Analysis (PCHA), we performed a simulation to assess the impact of outliers on these methods, as well as to find the critical point at which an outlier becomes a rare cell type. To this end, we again focus on the **CellLines** dataset. In this case, *H1* cell line (embryonic stem cell) is the farthest from the rest of cell lines. We set up an experiment in which we held out *H1* and gradually introduced different percentages of *H1* cells, varying from one to ten percent. For each case, we tried 10 individual replicas. Supplementary Figure 7a-c presents the performance of each method in identifying cell types, measured with respect to known cell types. In each case, we observe that preconditioning (Pre-SPA) significantly enhances the quality of results compared to Successive Projection Algorithm (SPA) alone. However, this makes the results unstable (has a high variance). Applying PCHA on top (PreSPA+PCHA) smooths out these variations. In order to assess the performance of these methods in identifying rare cell types, we used bipartite matching in each case to find the closest predicted cell type to *H1* and then used hypergeometric *p*-value to assess the overlap of these two sets. These results, presented in Supplementary Figure 7d, show that both PreSPA, and PreSPA+PCHA are sensitive enough to identify rare cell types. However, PreSPA is sensitive to low percentages of introduced *H1*, whereas PreSPA-PCHA considers percentages less than 2% to be noise/outlier and after that starts to identify it as a rare cell type.

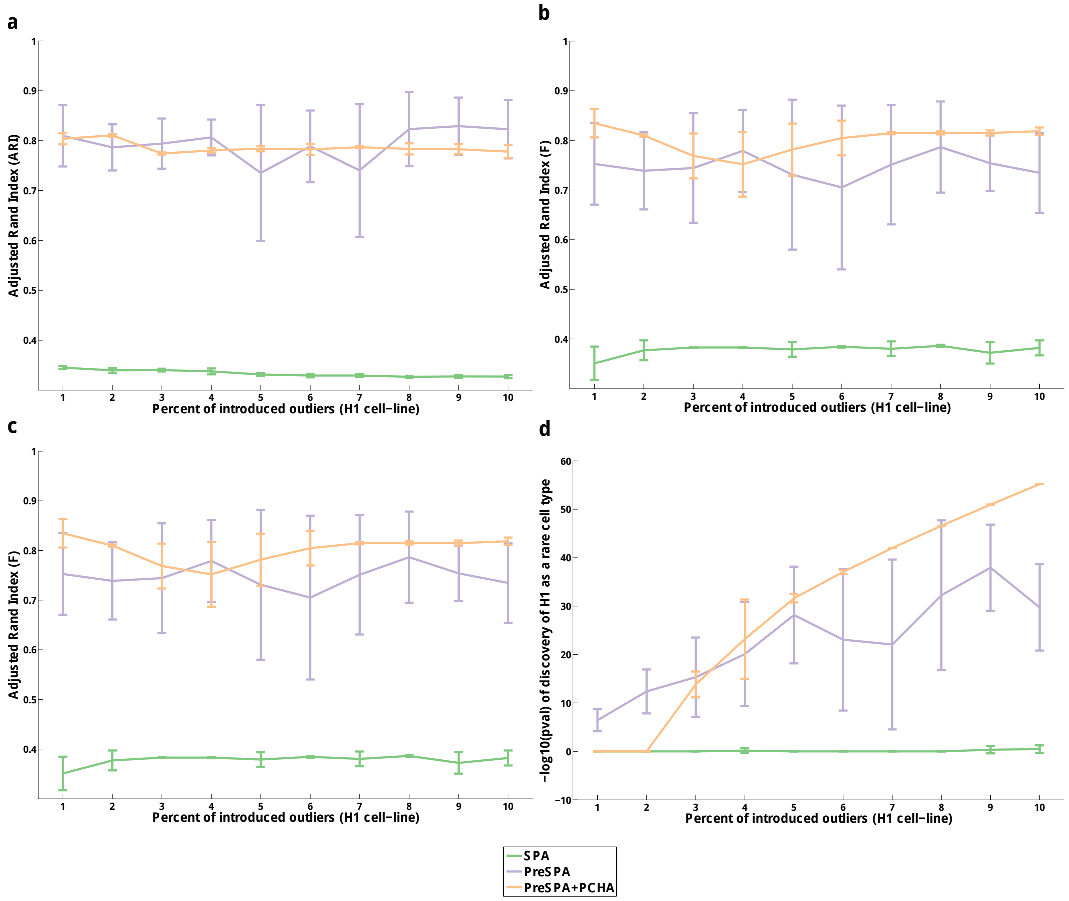

Supplementary Figure 7: **Robustness of *ACTION* method in presence of noise and outliers (a)-(c)** Different measures of cell type identification quality as a function of introduced noise, **(d)** Analysis of the critical point of transitioning from noise to rare cell type.

Supplementary Note 10: Visualizing the functional space of cells

Unlike the conventional application of t-distributed stochastic neighbor embedding (tSNE), which is used to project the transcriptional profile of cells into a lower dimensional space, we propose a framework that captures the distribution of cells around archetypes. To this end, we focus on the functional identity of cells with respect to our archetypes, which is computationally represented by the matrix $\mathbf{H}$. Each column in this matrix is a stochastic vector (sums to one) that represents the extent to which a cell is close to a given archetype. To visualize this continuous functional space, we first initialize the solutions using the Fiedler embedding (as opposed to tSNE that uses random initialization). Then, we use tSNE to update the initial coordinates. The following pseudo-code illustrates the proposed projection.

1. Take $\mathbf{H}$ from the PCHA as input.
2. Set $\tilde{\mathbf{H}} = [\mathbf{H}; \mathbf{I}]$
3. Let $\tilde{h}_i$ be the ith column of $\tilde{\mathbf{H}}$, and compute entries of the matrix $D_{ij} = \|\tilde{h}_i - \tilde{h}_j\|_2$ (that is, Euclidean distance between vectors $\tilde{h}_i$ and $\tilde{h}_j$).
4. Convert Distances to Similarity following Network Similarity Fusion [13] affinity matrix construction
   (a) Let d_i^* be the average distance from i^{th} cell to its top $k = \mathbf{round}(n/10)$ closest neighbors, with n being the total number of cells. (If you sort columns of the matrix $\mathbf{D}$, this is just the top k entries.)
   (b) Set $\Sigma_{i,j} = (d_i^* + d_j^* + 2\varepsilon + D_{ij})/3$, where ε is 2^{-52}.

(c) Set $\tilde{\Sigma}_{i,j} = \begin{cases} \Sigma_{i,j} + \varepsilon & \Sigma_{i,j} \geq \varepsilon \\ \varepsilon & \text{Otherwise.} \end{cases}$

(d) Set $W_{i,j}$ to be the probability that a normally distributed random variable with mean 0 and standard deviation $\tilde{\Sigma}_{i,j}$ has value $D_{i,j}$.

5. Set $\mathbf{G} = (\mathbf{W} + \mathbf{W}^T)/2$ be the weighted graph between cells.

6. Set $\mathbf{L} = \text{diag}(\mathbf{G} \cdot \text{ones}(n, 1)) - \mathbf{G}$ (that is, $\mathbf{L}$ is the combinatorial Laplacian of $\mathbf{G}$).

7. Compute the three smallest eigenvalues and eigenvectors of $\mathbf{L}$, $(\boldsymbol{v}_1, \lambda_1), (\boldsymbol{v}_2, \lambda_2), (\boldsymbol{v}_3, \lambda_3)$. Note that λ_1 is zero because of the Laplacian structure.

8. Set $\boldsymbol{x} = \boldsymbol{v}_2/\sqrt{\lambda_2}$

9. Set $\boldsymbol{y} = \boldsymbol{v}_y/\sqrt{\lambda_3}$

10. Run t-SNE to update $\boldsymbol{x}, \boldsymbol{y}$ coordinates.

11. Final map represents the distribution of cells around each archetype.

Supplementary Note 11: List of 20 top-ranked genes for each archetype in the Melanoma dataset

1-T	2-B	3-T/Unresolved	4-Tumor	5-Tumor	6-Tumor	7-Macro	8-Endo/CAF
**NKG7**	**MS4A1**	UGDH-AS1	DCT	APOC2	SAA1	**TYROBP**	IGFBP7
**CD8A**	**CD79A**	ROCK1P1	**PMEL**	APOD	TF	**FCER1G**	EFEMP1
**CST7**	HLA-DRA	TMEM212	LHFPL3-AS1	**SERPINA3**	SFRP1	**CD14**	CCL21
GZMA	**BANK1**	HERC2P4	CTSK	**MIA**	MAGEA4	**IFI30**	BGN
**CD3D**	**CD79B**	ASTN2	TYRP1	A2M	RGS5	**C1QC**	**THY1**
CCL4	IGLL5	SHISA9	TUBB4A	**SERPINE2**	MAGEC2	**C1QA**	TFPI
**IL32**	**IRF8**	ORC4	SCD	**TYR**	PDK4	**AIF1**	CLDN5
**GZMK**	**CD37**	SPC25	**GPM6B**	APOE	C2orf82	**C1QB**	PDLIM1
**PRF1**	**CD19**	LOC643406	**RAB38**	IFI27	ALDH1A3	**S100A9**	**COL1A1**
**CD2**	CD74	LOC646214	**PIR**	TRIML2	CAMP	**FCGR3A**	**C1R**
**KLRK1**	CXCR4	ODF2L	KIT	MFGE8	**SERPINA3**	**CSF1R**	**RARRES2**
ITM2A	SELL	ABCC9	CA14	NSG1	C1QTNF3	**MS4A6A**	CYR61
RGS1	**VPREB3**	L2HGDH	BCAN	RDH5	ANGPTL4	**IGSF6**	**DCN**
PDCD1	HLA-DPA1	LYZ	SNAI2	**SLC26A2**	ERRFI1	**HCK**	**C1S**
CD27	TCL1A	LOC286437	GSTO1	**CAPN3**	COL1A2	**PILRA**	NNMT
**TIGIT**	HLA-DQB1	MAB21L3	**MLANA**	MT2A	MRPL36	**VSIG4**	CXCR7
**LCK**	**BCL11A**	KCNQ1OT1	**SLC45A2**	SPP1	FN1	IL1B	IGFBP4
CTSW	LTB	ARHGEF26-AS1	**GPR143**	TM4SF1	HAPLN1	TMEM176B	ECSCR
IL2RG	NAPSB	FBLIM1	TRPM1	**PMEL**	SAA2	**CD163**	GNG11
**SIRPG**	**CD22**	GLIPR1L2	**CDK2**	**CDH19**	MGP	FCN1	CLU
7.7×10^{-67}	3.5×10^{-59}	1.6×10^{-3}	2.5×10^{-22}	4.8×10^{-53}	5.0×10^{-05}	2.4×10^{-176}	5.9×10^{-98}

Supplementary Table 1: Table of the top 20 residual genes after orthogonalization. Each archetype is also annotated with its enriched cell type. Bolded genes are the genes that coincide with known markers provided by the original paper. The last row is the p-value of enrichment of markers among all genes sorted after orthogonalizing each archetype.

Supplementary Note 12: Regulated downstream targets of MITF factor in Subclasses *B* and *C*

The following table lists the full set of significant downstream targets of MITF in both subclasses *B & C*. Genes *GPNMB, MLANA, PMEL* and *TYR* are shared between two subclasses, whereas the rest of targets are unique to one of them. For genes that have significant effect on the survival rate, their Cox coefficient is presented in the table. A positive Cox coefficient indicates that high expression of the given genes is associated with poor survival.

Target	Subclass B	Subclass C	Cox Coefficient
ACP5	✓		-
CDK2	✓		0.218
CTSK	✓		-
DCT	✓		-
KIT	✓		0.3214
OCA2	✓		0.3038
TRPM1	✓		0.188
TYRP1	✓		0.2422
GPNMB	✓	✓	-
MLANA	✓	✓	-
PMEL	✓	✓	0.2765
TYR	✓	✓	-
BEST1		✓	-
BIRC7		✓	-
FOS		✓	-
MET		✓	-

Supplementary Table 2: MITF target genes in Subclasses $B\&C$

Supplementary References

1. Xu, C. & Su, Z. Identification of cell types from single-cell transcriptomes using a novel clustering method. *Bioinformatics* **31**, 1974–1980 (2015).

2. Satija, R., Farrell, J. A., Gennert, D., Schier, A. F. & Regev, A. Spatial reconstruction of single-cell gene expression data. *Nature Biotechnology* **33**, 495–502 (2015).

3. Ji, Z. & Ji, H. TSCAN: Pseudo-time reconstruction and evaluation in single-cell RNA-seq analysis. *Nucleic Acids Research* **44**, e117–e117 (2016).

4. Marco, E. *et al.* Bifurcation analysis of single-cell gene expression data reveals epigenetic landscape. *Proceedings of the National Academy of Sciences* **111**, E5643–E5650 (2014).

5. Zeisel, A. *et al.* Cell types in the mouse cortex and hippocampus revealed by single-cell RNA-seq. *Science* **347**, 1138–42 (2015). 9809069v1.

6. Grün, D. *et al.* Single-cell messenger RNA sequencing reveals rare intestinal cell types. *Nature* **525**, 251–5 (2015).

7. Jiang, L., Chen, H., Pinello, L. & Yuan, G.-C. GiniClust: detecting rare cell types from single-cell gene expression data with Gini index. *Genome Biology* **17**, 144 (2016).

8. Haghverdi, L., Buettner, F. & Theis, F. J. Diffusion maps for high-dimensional single-cell analysis of differentiation data. *Bioinformatics* **31**, 2989–2998 (2015).

9. Korem, Y. *et al.* Geometry of the Gene Expression Space of Individual Cells. *PLOS Computational Biology* **11**, e1004224 (2015).

10. Hart, Y. *et al.* Inferring biological tasks using Pareto analysis of high-dimensional data. *Nature Methods* **12**, 233–235 (2015).

11. Shoval, O. *et al.* Evolutionary Trade-Offs, Pareto Optimality, and the Geometry of Phenotype Space. *Science* **336**, 1157–1160 (2012).

12. Gillis, N. & Vavasis, S. A. Semidefinite Programming Based Preconditioning for More Robust Near-Separable Nonnegative Matrix Factorization. *SIAM Journal on Optimization* **25**, 677–698 (2015).

13. Wang, B. *et al.* Similarity network fusion for aggregating data types on a genomic scale. *Nature Methods* **11**, 333–337 (2014).
